# Association between sarcopenic obesity and dementia in the Chinese elderly using different definitions of obesity: evidence from the CHARLS

**DOI:** 10.3389/fnagi.2025.1540272

**Published:** 2025-06-04

**Authors:** Lang Peng, Qingwei Xiang, Ge Jia, Renyi Yin

**Affiliations:** ^1^Department of Geriatrics, Hubei Provincial Hospital of Traditional Chinese Medicine, Wuhan, China; ^2^Affiliated Hospital of Hubei University of Chinese Medicine, Wuhan, China; ^3^Hubei Provincial Academy of Traditional Chinese Medicine, Wuhan, China; ^4^Wuhan No.1 Hospital, Wuhan, China

**Keywords:** sarcopenic obesity, dementia, cross-sectional study, probable sarcopenia, CHARLS

## Abstract

**Objectives:**

The prevalence of sarcopenic obesity, characterized by the coexistence of reduced muscle mass and function alongside increased adipose tissue, is increasing in the aging population. This study aims to investigate the association between sarcopenic obesity and dementia risk in community-dwelling older adults in China, utilizing a nationally representative dataset. Furthermore, we aim to assess the comparative effectiveness of waist circumference versus body mass index as indicators for assessing this risk association.

**Design:**

The study was designed as a cross-sectional study.

**Setting and participants:**

Based on the nationally representative data from the China Health and Retirement Longitudinal Study (CHARLS) 2015, a total of 5,320 community-dwelling participants aged 60 years and above were categorized into four groups according to their respective classifications of normal status, possible sarcopenia, obesity, and sarcopenic obesity.

**Methods:**

Multivariable logistic regression models were used to analyze the relationship between sarcopenic obesity and probable dementia while adjusting for potential confounders.

**Results:**

After adjusting for potential confounders, possible sarcopenia alone (*OR* 1.674, *95% CI* 1.238–2.264) and sarcopenic obesity (*OR* 1.812, *95% CI* 1.325–2.479) were significantly associated with an increased risk of probable dementia. In contrast, abdominal obesity alone, defined by waist circumference (WC), was not significantly associated with dementia risk. When stratified by age and gender, the association between sarcopenic obesity and probable dementia remained significant.

**Conclusion and implications:**

The findings from this cross-sectional study suggest that both possible sarcopenia and sarcopenic obesity are significantly associated with an increased risk of probable dementia among older adults residing in the community in China. Notably, the relationship between sarcopenic obesity and dementia appears to be more pronounced compared to either possible sarcopenia or obesity alone. Moreover, incorporating waist circumference alongside components of possible sarcopenia may serve as a more effective predictor of cognitive impairment when compared to relying solely on body mass index (BMI). These results underscore the critical importance of early identification and intervention for individuals with sarcopenic obesity to mitigate the risk of developing dementia.

## Introduction

Sarcopenic obesity is defined as a functional and clinical condition characterized by a concurrent decline in muscle mass and function, along with increased adipose tissue (Carla et al., 2024; Shibo et al., 2023). The incidence of sarcopenic obesity is increasing rapidly, mainly due to the aging global population and the current obesity epidemic (Jing et al., 2024). A meta-analysis of 50 studies, representing 86,285 individuals, indicated that sarcopenic obesity affects more than one in 10 older adults worldwide (Qianqian et al., 2021). Sarcopenic obesity is considered a high-risk geriatric syndrome, which is of increasing concern as it is associated with significant health consequences, including implications for frailty, fractures, cognitive dysfunction, cardiovascular diseases, and an increased risk of hospitalization and mortality (Carla et al., 2024; Shibo et al., 2023; David et al., 2016; Simiao and Yang, 2015; Magdalena I et al., 2018; Minglan et al., 2024).

Dementia encompasses a range of neurodegenerative disorders that have a detrimental impact on cognitive functions, including memory, cognition, and affective processes. It has emerged as a pressing global public health priority for aging populations, presenting significant societal and economic challenges (Alzheimers Dement, 2023; Yuyang et al., 2024; Carol and Bruce, 2017). In the absence of curative treatments for dementia, there is significant interest in modifiable risk factors. Sarcopenia and obesity are both potential modifiable risk factors for alleviating the burden of cognitive impairment and dementia. Growing evidence suggests that sarcopenia and its components, particularly low handgrip strength (HGS), may be a good indicator of poor cognitive function and dementia (Ailing et al., 2023; Tao-Chun et al., 2020; Andrea et al., 2018; Michal et al., 2021). The association between obesity and cognitive impairment has been demonstrated in numerous epidemiological studies, although its establishment is not as robust compared to that of other comorbidities (Chan-Hee and Ji-Oh, 2022). The methodology used to define obesity may partially account for the uncertainty regarding whether obesity is a modifiable risk factor for dementia. Previous studies reported inconsistent results regarding the relationship between obesity, defined by body mass index (BMI), and cognitive function. Some studies have found a positive correlation between higher BMI and an increased risk of cognitive impairment (Gallucci et al., 2013; Emiliano et al., 2017; Kirsty et al., 2019), while others have observed a negative association between them (Nawab et al., 2015; Sujin et al., 2016; Ruo-Tong et al., 2024). Unfortunately, BMI fails to account for the distribution or quantity of adipose tissue and does not capture changes in body composition accurately. Waist circumference, a surrogate measure of visceral adipose tissue, has been examined in association with cognitive function. In general, some cross-sectional and prospective studies have suggested that increased central adiposity is associated with reduced cognitive functions (Theresia et al., 2023; Kazuaki et al., 2024; Nancy et al., 2017; Xingyao et al., 2021).

Recently, the special subtype of sarcopenia or obesity, marked by the coexistence of obesity and sarcopenia, sarcopenic obesity, has garnered attention regarding its association with cognitive function in the elderly. Several observational studies have suggested a potential association between sarcopenic obesity and a higher risk of cognitive impairment. Cross-sectional data from the Bunkyo Health Study and longitudinal data from the National Health and Aging Trends Survey (NHATS)—both of which divided participants according to low HGS and BMI-defined obesity—revealed that sarcopenic obesity was independently associated with an increased risk of impaired cognitive function in older adults (Yuki et al., 2022; John et al., 2021). Similarly, findings from the National Health and Nutrition Examination Survey (NHANES) 2011–2014 cohort, in which the sarcopenic obesity phenotypes were defined using HGS and waist circumference (WC), showed that individuals in the high WC-low HGS group had a higher risk of cognitive impairment (Uraiporn et al., 2024). Data from the UK Biobank showed that obesity, sarcopenia, and sarcopenic obesity were related to alterations in different brain regions (Junhan et al., 2024). However, it remains inconclusive whether sarcopenic obesity poses a greater risk for cognitive impairment than singular sarcopenia or obesity alone. Additionally, the diagnostic agreement for sarcopenic obesity varies considerably between different diagnostic methods of obesity, and waist circumference may be a more appropriate indicator for evaluating obesity among the Chinese population (Fengjuan et al., 2024). Considering large differences between different populations and geographic regions due to varying dietary habits (Jing et al., 2024), more relevant research is urgently needed, particularly in China, where the burden of cognitive disorders is dramatically increasing.

To address these gaps, data from the China Health and Retirement Longitudinal Study (CHARLS) were utilized in this cross-sectional study to explore the associations of sarcopenic obesity with dementia risk in community-dwelling older adults in China. In this study, we used WC as an obesity assessment indicator and investigated the differences between WC and BMI in identifying the relationship between sarcopenic obesity phenotypes and dementia.

## Methods

### Study design and participants

The study is based on data from the CHARLS, a national longitudinal cohort study on aging established in 2011 (Yaohui et al., 2012). The CHARLS employed a multistage, stratified, probability-proportional sampling method and utilized uniformly trained investigators to collect high-quality data via face-to-face interviews. These interviews gathered sociodemographic, lifestyle, and health-related information. Since the baseline survey in 2011–2012, follow-up visits have been conducted biennially for the individuals involved. The CHARLS was ethically approved by the Institutional Review Board at Peking University. All participants provided signed informed consent. Details are reported on the CHARLS website.^1^

**Figure 1:**
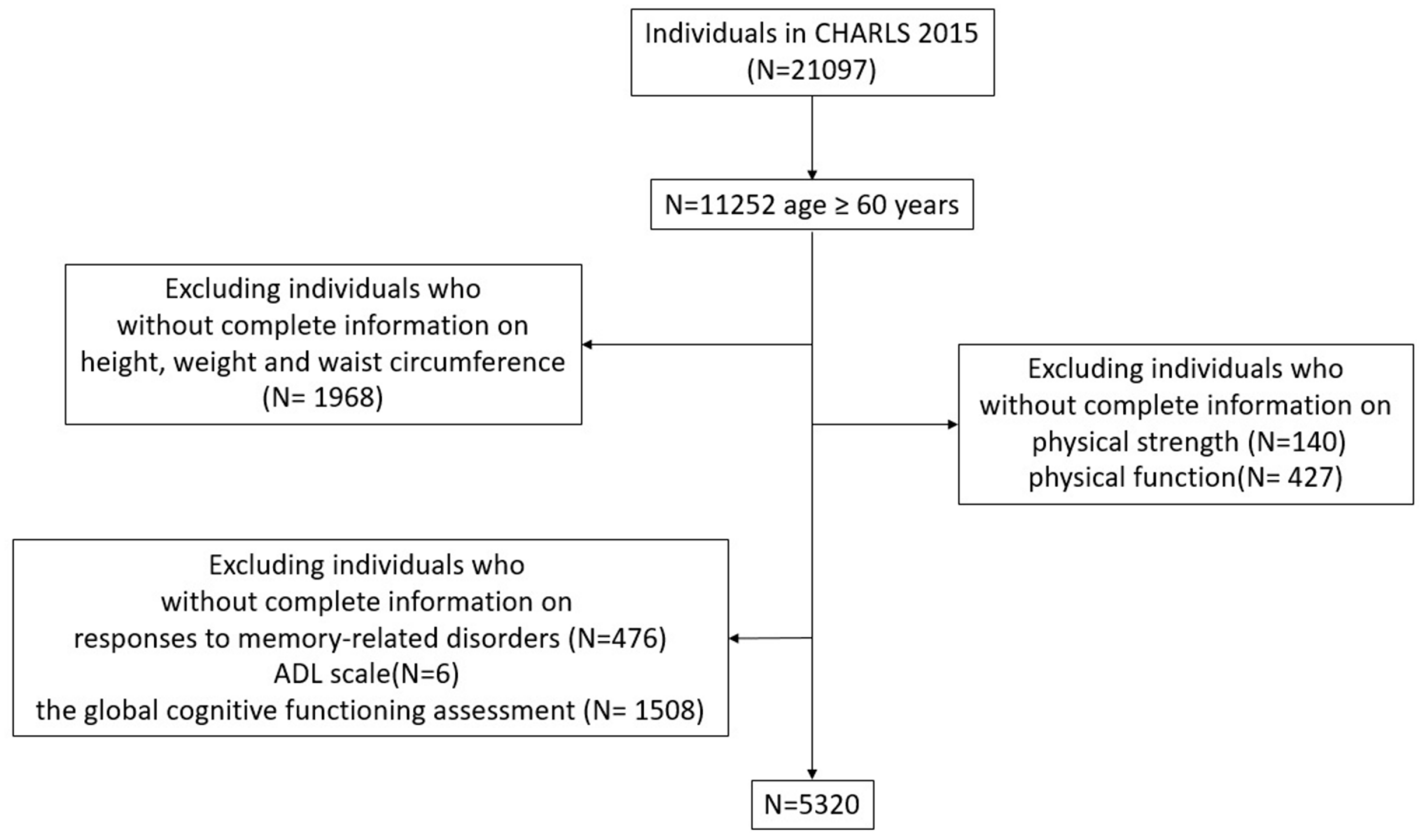
Flowchart of participant enrolment.

This cross-sectional study utilized data from the 2015 wave of the CHARLS, during which the natural aging of the original cohort and sample replenishment substantially increased the number of participants aged 60 years and older. The analysis included individuals aged ≥60 with complete data on possible sarcopenia, obesity, and probable dementia diagnoses. A total of 21,097 individuals were involved at baseline, of which 15,777 were excluded for the following reasons: age less than 60 years (*n* = 9,845), a lack of obesity-related data (*n* = 1968), a lack of sarcopenia information (*n* = 567), and a lack of data on probable dementia (*n* = 1990). Ultimately, our study involved a total of 5,320 participants. The flow path of the inclusion and exclusion process is presented in Figure 1.

### Primary predictor variables

Although skeletal muscle strength and mass are still considered fundamental to a definitive clinical diagnosis, the Asian Working Group for Sarcopenia (AWGS) 2019 introduced “possible sarcopenia,” defined by either low muscle strength or low physical performance, to enable earlier lifestyle interventions in the community environment (Liang-Kung et al., 2020). In the present study, the participants are defined as having “possible sarcopenia” when they exhibit low muscle strength or low physical performance, according to AWGS 2019. Low muscle strength is defined by HGS < 28 kg for men and <18 kg for women; the criterion for low physical performance is a 5-time chair stand test ≥12 s. HGS was tested using a mechanical dynamometer (Leapfrog TM WL-1000, Nantong, China). After the interviewer demonstrated how to perform the activity and proper arm position, the participants squeezed as hard as possible, and the value was recorded to the nearest 10th of a kilogram. The maximum HGS value was taken from two measurements for each hand. The 5-time chair stand test measured the time it takes for participants, seated on a standard stool with arms crossed over their chest, to stand up and sit down five times as quickly as possible without using their hands.

Sarcopenic obesity was defined as the coexistence of possible sarcopenia and obesity, determined by either waist circumference (WC ≥ 90 cm for men and ≥85 cm for women) (Ling et al., 2024) or body mass index (BMI > 28.0 kg/m^2^) (Dandan et al., 2021). The WC was measured using a flexible tape measure on the participant’s abdomen aligned with their navel in a snug, but not tight manner, holding their breath at the end of exhalation, and the WC was recorded in centimeters. BMI was derived by dividing the respondents’ measured weight (kg) by the squared value of their measured height (m).

We divided participants into four groups according to their possible sarcopenia and obesity status as normal, possible sarcopenia, obesity, and sarcopenic obesity. Specifically, Sarcopenic obesity was defined as participants fulfilling definitions of both sarcopenia and obesity; normal was defined as participants fulfilling neither sarcopenia nor obesity. Participants who met the criteria for possible sarcopenia but not obesity were categorized into the possible sarcopenia group, and those who were obese but not possible sarcopenia were categorized into the obesity group.

### Assessment of probable dementia

In the CHARLS, participants were asked whether they were diagnosed with memory-related diseases, which included not only dementia but also brain atrophy and Parkinson’s disease. Therefore, we defined probable dementia with the operational criteria used in the English Longitudinal Study of Aging (Sara et al., 2017). Briefly, objective cognitive function tests, informant-reported cognitive status of respondents, and functional status were used together to identify those with probable dementia. In the CHARLS 2015, the global objective cognitive function tests were administered, covering the domains of concentration, memory function, verbal fluency, and executive function. Those with impairment in two or more domains (defined as scores of 1.5 standard deviations below the mean or lower within each education group) were considered to have cognitive impairment. Functional status was measured by activities of daily living (ADL), and difficulty in performing any one of the ADL was defined as functional impairment. Probable dementia was defined either as a combination of cognitive impairment and functional impairment or an informant-reported diagnosis of memory-related diseases.

### Covariates

The following baseline characteristics were identified as covariates in our study, mainly involving sociodemographic factors, lifestyles, and health conditions. Demographic factors included age (years), sex (female or male), residence (urban or rural), marital status (married and partnered or others), and education (less than lower secondary, upper secondary, vocational training, or tertiary). Lifestyles comprised drinking status (never, ever) and smoking status (never, ever). Health conditions consisted of physician-diagnosed history of hypertension, diabetes, heart-related diseases, stroke, cancer, and dyslipidemia. Depressive symptoms were evaluated according to the Center for Epidemiologic Studies Depression (CES-D) scale, and a CES-D score ≥10 was considered depressive symptoms. All covariates have been reported to be related to cognitive function or dementia risk (Sebastian et al., 2024).

### Statistical analysis

Data were presented as mean and standard deviation (SD) or median and interquartile range (IQR) for continuous variables, and as frequency (percentage) for categorical variables. Differences in descriptive characteristics across the four groups were examined using one-way ANOVA or the Kruskal–Wallis test for continuous variables and chi-squared tests for categorical variables. Separate multivariable logistic regression models were used to analyze the association between possible sarcopenia, obesity, and sarcopenic obesity with probable dementia (reference: the normal group). The crude model did not adjust for covariates; in the multivariate-adjusted model, we adjusted for age, gender, marital status, education level, residential status, drinking status, smoking status, hypertension, diabetes, heart-related diseases, stroke, cancer, hyperlipidemia, and depressive symptoms. We conducted several sensitivity analyses to test the robustness of findings: (Carla et al., 2024) Considering the differing progression of obesity and possible sarcopenia in men and women (Karthickeyan et al., 2021), as well as the close relationship between dementia and age, we conducted subgroup analyses by age (i.e., less than 75 years old and 75 years old or above) and gender. (Shibo et al., 2023) Based on the components of possible sarcopenia, we analyzed the association between different possible sarcopenia components combined with obesity, defined by waist circumference, and the risk of dementia separately. Finally, we redefined sarcopenic obesity (SO) according to possible sarcopenia with BMI-defined obesity, and we investigated the differences between WC and BMI when identifying the relationship between SO phenotypes and dementia.

All analyses were conducted with SPSS V.26 (IBM, Armonk, NY, USA). All *p-values* were two-tailed, and a *p*-value of < 0.05 was considered to be statistically significant.

## Results

The analytic sample included 5,320 participants in the age range of 60–105 years, and their descriptive characteristics are listed in Table 1. A total of 2,973 (55.9%) were male, and the mean age was 67.20 ± 5.88 years. Overall, 872 (16.4%) participants were classified as having possible sarcopenia alone, 1,623 (30.5%) with obesity alone, 759 (14.3%) with sarcopenic obesity, and 2,066 (38.8%) with neither condition. Participants identified with possible sarcopenia alone were predominantly female (64.9%), while among 759 individuals identified with sarcopenic obesity, there were 487 women (64.2%). In comparison to participants in the normal group, individuals with possible sarcopenia or sarcopenic obesity were more likely to be older, live in rural areas, and have a lower level of education, while those with obesity were more likely to be younger, live in urban areas, and have a higher level of education. In comparison to participants with sarcopenia alone, individuals with sarcopenic obesity were more likely to be female, never smoke, never drink, and more likely to have hypertension, diabetes, dyslipidemia, heart problems, and stroke. There were 464 (8.72%) probable dementia cases, with the possible sarcopenia group and the SO group having 116 (6.9%) and 117 (15.4%) cases of probable dementia patients, respectively.

**Table 1:** Baseline characteristics of the study participants according to the possible sarcopenia and circumference–defined obesity status.

Characteristics	Normal (*n* = 2,066)	Possible Sarcopenia (*n* = 872)	Abdominal obesity (*n* = 1,623)	Sarcopenic obesity (*n* = 759)	*p*-value
Age, years	66.44 (5.33)	70.07 (6.75)	65.88 (5.06)	68.83 (6.33)	*p* < 0.001
Sex, *n* (%)					
Male	1,423 (68.9)	566 (64.9)	712 (43.9)	272 (35.8)	*p* < 0.001
Female	643 (31.1)	306 (35.1)	911 (56.1)	487 (64.2)	
Residence, *n* (%)					
Urban	708 (34.3)	228 (26.1)	838 (51.6)	335 (44.1)	*p* < 0.001
Rural	1,358 (65.7)	644 (73.9)	785 (48.4)	424 (55.9)	
Education level, *n* (%)					
Less than lower secondary	1,905 (92.2)	830 (95.2)	1,454 (89.6)	720 (94.9)	*p* < 0.001
Upper secondary and vocational training	130 (6.3)	32 (3.7)	127 (7.8)	28 (3.7)	
Tertiary	31 (1.5)	10 (1.1)	42 (2.6)	11 (1.4)	
Marital status, *n* (%)					
Married/Partnered	1,776 (86.0)	665 (76.3)	1,387 (85.5)	585 (77.1)	*p* < 0.001
Others^1^	290 (14.0)	207 (23.7)	235 (14.5)	174 (22.9)	
Smoking, *n* (%)					
Never smoker	809 (39.2)	337 (38.6)	960 (59.1)	476 (62.7)	*p* < 0.001
Ever smoker	1,257 (60.8)	535 (61.4)	663 (40.9)	283 (37.3)	
Drinking, *n* (%)					
Never drinker	879 (42.6)	408 (46.8)	871 (53.7)	465 (61.3)	*p* < 0.001
Ever drinker	1,185 (57.4)	463 (53.2)	750 (46.3)	293 (38.7)	
BMI (*kg/m^2^*)	21.55 (3.05)	20.75 (2.41)	26.28 (3.51)	26.33 (3.99)	*p* < 0.001
Waist circumference, *cm*	76.87 (12.19)	76.82 (11.47)	94.91 (6.47)	95.85 (6.91)	*p* < 0.001
Comorbidities, *n* (%)					
Hypertension	554 (27.1)	289 (33.8)	771 (48.1)	427 (56.9)	*p* < 0.001
Diabetes	133 (6.5)	70 (8.1)	246 (15.5)	145 (19.5)	*p* < 0.001
Dyslipidemia	263 (13.2)	119 (14.2)	446 (28.3)	252 (34.3)	*p* < 0.001
Cancer	30 (1.5)	17 (2.0)	23 (1.4)	15 (2.0)	*p* = 0.580
Heart problem	314 (15.3)	183 (21.2)	428 (26.7)	225 (30.0)	*p* < 0.001
Stroke	55 (2.7)	45 (5.2)	63 (3.9)	49 (6.5)	*p* < 0.001
Depressive symptoms	619 (30.0)	370 (42.5)	428 (26.4)	309 (40.7)	*p* < 0.001
Probable dementia, *n* (%)	119 (5.8)	116 (13.3)	112 (6.9)	117 (15.4)	*p* < 0.001

1 Others included separated, divorced, widowed, and never married.

Table 2 shows different associations of possible sarcopenia, obesity, and sarcopenic obesity with probable dementia. If the definition of abdominal obesity is adopted, in the unadjusted and multivariable-adjusted models, compared to participants in the normal group, those with possible sarcopenia or sarcopenic obesity were associated with an elevated probable dementia risk. Relative to the normal group, those with possible sarcopenia alone were associated with 1.674 (95% confidence interval [*CI*]: 1.238–2.264) greater odds for probable dementia, while those with abdominal obesity alone were not statistically associated with probable dementia (*OR* = 1.045; *95%CI*: 0.773–1.412). Those with sarcopenic obesity had the highest odds for probable dementia (*OR* = 1.812, *95%CI*: 1.325–2.479) in fully adjusted models (*p* < 0.001), compared to the normal group. If BMI-defined obesity is adopted, our results indicated that possible sarcopenia alone is statistically associated with probable dementia, with an *OR* of 1.807 (*95% CI*: 1.433–2.279), whereas the coexistence of possible sarcopenia and obesity based on the BMI criteria showed no significant correlation with dementia, with an *OR* of 1.286 (*95% CI*: 0.773–2.141), relative to the normal group.

**Table 2:** Associations of possible sarcopenia, obesity, and sarcopenic obesity with probable dementia.

Groups	Crude model		Adjusted model	
	OR (95% Cl)	*p*-value	OR (95% Cl)	*p*-value
Adopt the definition of abdominal obesity				
Normal	Ref		Ref	
Possible sarcopenia alone	**2.510 (1.918–3.286)**	***p* < 0.001**	**1.674 (1.238–2.264)**	***p* = 0.001**
Abdominal Obesity alone	1.213 (0.929–1.583)	*p* = 0.156	1.045 (0.773–1.412)	*p* = 0.776
Sarcopenic obesity^1^	**2.982 (2.275–3.907)**	***p* < 0.001**	**1.812 (1.325–2.479)**	***p* < 0.001**
Adopt the BMI definition of obesity				
Normal	Ref	–	Ref	**–**
Possible sarcopenia alone	**2.609 (2.127–3.199)**	***p* < 0.001**	**1.807 (1.433–2.279)**	***p* < 0.001**
BMI-defined obesity alone	1.218 (0.803–1.847)	*p* = 0.354	1.175 (0.741–1.864)	*p* = 0.493
Sarcopenic obesity^2^	**2.121 (1.353–3.326)**	***p* = 0.001**	1.286 (0.773–2.141)	*p* = 0.333

Adjusted model: Adjusted for age, sex, educational attainment, marital status, residence, cigarette smoking status, drinking status, hypertension, diabetes, dyslipidemia, cancer, heart problem, stroke, and depressive symptoms.

OR, odds ratio; CI, confidence interval.

1 Sarcopenic obesity was defined as the coexistence of possible sarcopenia and abdominal obesity.

2 Sarcopenic obesity was defined as the coexistence of possible sarcopenia and BMI-defined obesity.

Boldface numbers indicate statistical significance.

**Figure 2:**
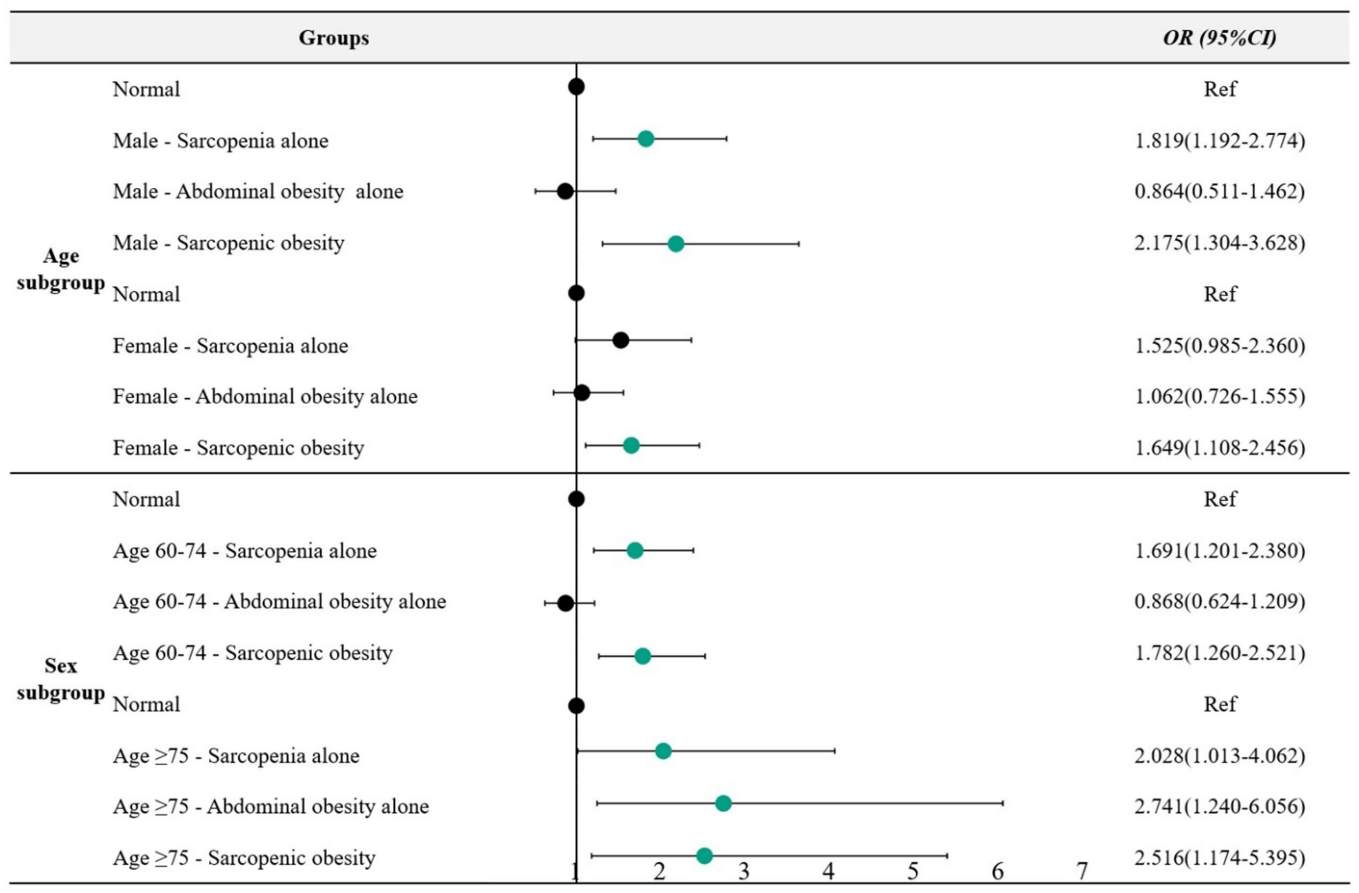
Associations of possible sarcopenia, abdominal obesity, and sarcopenic obesity with probable dementia stratified by age and sex. Adjusted model 1: Adjusted for age, educational attainment, marital status, residence, cigarette smoking status, drinking status, hypertension, diabetes, dyslipidemia, cancer, heart problem, stroke, and depressive symptoms. Adjusted model 2: Adjusted for sex, educational attainment, marital status, residence, cigarette smoking status, drinking status, hypertension, diabetes, dyslipidemia, cancer, heart problem, stroke, and depressive symptoms. OR, odds ratio; CI, confidence interval.

We conducted stratified analyses by age and sex based on the multivariable-adjusted model (Figure 2). In the sex-stratified analysis, compared to the normal group, male participants with possible sarcopenia alone and sarcopenic obesity exhibited significantly higher odds of probable dementia (1.819 [*95%CI*: 1.192–2.774] and 2.175 [*95%CI*: 1.304–3.628]), respectively. In contrast, among all female participants, a statistically significant association was observed only between sarcopenic obesity and probable dementia (*OR* = 1.649, *95%CI*: 1.108–2.456), and the relationship between possible sarcopenia alone and probable dementia suggested a trend toward increased that warrants further investigation (*OR* = 1.525, *95%CI*: 0.985–2.360; *p* = 0.058), relative to the normal group. In the age-stratified analysis, individuals aged 60–74 years with possible sarcopenia alone exhibited 1.691 greater odds for probable dementia (*95% CI*: 1.201–2.380), and those with sarcopenic obesity exhibited 1.782 greater odds for probable dementia (*95% CI*: 1.260–2.521), relative to the normal group. In individuals aged 75 years and older, those with possible sarcopenia alone or sarcopenic obesity had higher odds for probable dementia, with odds ratios of 2.028 (*95% CI*: 1.013–4.062) and 2.516 (*95% CI*: 1.174–5.395) compared to the normal group of the same age range, respectively. Furthermore, individuals aged 75 years and older with abdominal obesity alone were associated with 2.741 (*95% CI*: 1.240–6.056) greater odds for probable dementia, relative to the normal group of the same age range.

We explored the associations of the components of possible sarcopenia in conjunction with obesity and probable dementia. We found that both low handgrip strength and low physical performance are closely related to dementia, with *OR* of **1.753** (*95% CI*: **1.260–2.439**) and 1.511 (*95%CI*: **1.090–2.094**) compared to the normal group. The coexistence of either low muscle strength or low physical performance with abdominal obesity further increases the risk of probable dementia, with *OR* of **1.617** (*95%CI*: **1.053–2.483**) and 1.891 (*95% CI*: 1.381–2.589), compared to the normal group, respectively.

## Discussion

We ascertained the relationship between possible sarcopenia, obesity, and sarcopenic obesity with the risk of probable dementia in a representative community-based elderly Chinese population. Our study shows that both possible sarcopenia and sarcopenic obesity lead to an increased risk of impaired cognitive function in the elderly. The sarcopenic obesity, coexistence of possible sarcopenia and obesity, was significantly associated with the greatest risk of dementia than singular possible sarcopenia or obesity. The impact of possible sarcopenia in the aging population is compounded by obesity, particularly abdominal obesity. Our findings are consistent with those of several recently published studies, which concluded that the SO phenotype was associated with an increased risk of cognitive dysfunction in older adults. The Bunkyo Health Study in Japan showed that sarcopenic obesity, defined by high BMI-low HGS, was independently associated with mild cognitive impairment and dementia, respectively (Yuki et al., 2022). In the 2011–2014 NHANES cohort, the high WC-Low HGS group showed significantly lower scores on all four cognitive tests and a higher risk of cognitive impairment (Uraiporn et al., 2024). The longitudinal data from the NHATS revealed that sarcopenia is associated with an elevated long-term risk of cognitive function impairment in older adults. However, no significant interaction term was found between sarcopenia and obesity (John et al., 2021). Our study expands on their previous reports and provides additional evidence that not only low HGS, but also low physical performance alone, is independently associated with probable dementia, and the combination of low physical performance and abdominal obesity is independently associated with probable dementia. In participants aged 75 or older, possible sarcopenia, abdominal obesity, and sarcopenic obesity are all independently associated with dementia.

Some studies showed sex differences in the association between obesity and dementia. Hu et al. found that BMI and hip circumference are positively associated with cognitive function in elderly women, a correlation not observed in elderly men (Yuxin et al., 2021). Guo et al. discovered that greater WC was positively correlated with enhanced cognitive function in elderly low-income men living in rural China, while a higher BMI was associated with an increased risk of cognitive impairment among elderly women (Dandan et al., 2021). In our study, male participants with sarcopenia alone or sarcopenic obesity exhibited significantly higher odds of probable dementia. In contrast, among female participants, only sarcopenic obesity was statistically associated with probable dementia. In the 60–74 age subgroup, individuals with sarcopenia or sarcopenic obesity had a significantly greater risk of dementia compared to those with normal muscle mass, and this risk increased with advancing age. Notably, in participants aged 75 and older, all three phenotypes—sarcopenia, sarcopenic obesity, and abdominal obesity—were associated with an elevated risk of dementia, with abdominal obesity showing the most pronounced increase in risk.

Previous studies on the association between obesity and cognitive function have shown inconsistent results. Some reported that a higher BMI tended to be a risk factor for cognitive impairment (Gallucci et al., 2013; Emiliano et al., 2017; Kirsty et al., 2019), while others observed a reduced risk of a higher BMI in cognitive decline (Nawab et al., 2015; Sujin et al., 2016; Ruo-Tong et al., 2024). The possible reasons for the differences in these studies may be related to different criteria for evaluating obesity and not further identifying the specific subtype of sarcopenic obesity. In our study, sarcopenic obesity is significantly related to dementia in the elderly, while there was a statistical association only observed between a large WC and dementia in those aged 75 and above, but this phenomenon was not observed in elderly individuals with a high BMI score, although BMI continues to be used as a common measure in clinical settings. Our findings are consistent with some studies, which suggested that a greater WC is associated with a greater rate of cognitive decline (Deckers et al., 2017; Leian et al., 2024; Diana et al., 2011), whereas BMI does not have such a relationship (Nancy et al., 2017).

Sarcopenic obesity in the elderly reflects age-related changes in body composition as well as the association between visceral fat and metabolic dysregulation. Common mechanisms believed to accelerate cognitive changes that lead to dementia involve a complex interplay between insulin resistance, oxidative damage, inflammation, cardiometabolic, and lifestyle factors, among others (Carla et al., 2024; Uraiporn et al., 2024; John and Dennis, 2018; Robert et al., 2014). Obesity, possible sarcopenia, and sarcopenic obesity were, respectively, related to alterations in different brain regions (Junhan et al., 2024); these obesity-associated changes in the brain are associated with cognitive dysfunction. Leonie Lampee et al. indicate an increase in white matter hyperintensities burden selectively in the deep white matter in obese subjects with high visceral fat accumulation (Leonie et al., 2019). In our study, patients with sarcopenic obesity exhibited a higher prevalence of hypertension, diabetes, dyslipidemia, heart disease, stroke, and depressive symptoms compared to those with abdominal obesity. These conditions are well-documented risk factors for dementia, suggesting a potential mechanistic link between sarcopenic obesity and cognitive decline.

## Conclusion and implications

Both sarcopenic obesity and possible sarcopenia are associated with a higher risk of impaired cognitive function, and the association between sarcopenic obesity and dementia is strengthened due to the interaction between abdominal obesity and low HGS or low physical performance. The combination of possible sarcopenia and waist circumference, rather than BMI, might be more predictive of cognitive performance than either measure alone in a nationally representative sample in China. This analysis provides empirical evidence that supports the necessity for identifying individuals who exhibit both possible sarcopenia and sarcopenic obesity in clinical practice. Waist circumference management and exercises for muscle strength and function in the elderly population in China may have cognitive benefits.

## Limitations

Some limitations of this study should be acknowledged. The estimates may have been influenced by biases in self-reported information on covariates. Despite careful adjustments for covariates, there remains a concern regarding potential residual confounding effects, particularly related to factors such as the lack of dementia-related biomarkers (e.g., APOE ε4) and physical activity due to the presence of missing or incomplete data in the CHARLS dataset, which could impact the observed associations. Given the cross-sectional nature of our research design, causal conclusions cannot be drawn. Additionally, the definition of dementia and sarcopenia was measured in an unstandardized way. However, ways of utilizing the operational criteria and self- or proxy-reported diagnosis to define dementia were both widely used and validated in epidemiological studies. Although muscle mass should be evaluated as a component in defining sarcopenia, the inability to perform an accurate assessment using dual-energy X-ray absorptiometry or multi-frequency bioelectrical impedance analysis in cost-effective clinical practice continues to be problematic. AWGS 2019 introduced “possible sarcopenia,” which has also been extensively applied in epidemiological research. Therefore, further prospective studies are needed to investigate potential causal or bidirectional relationships between sarcopenic obesity and dementia, based on more rigorous definitions. Multicomponent interventional studies targeting elements of both sarcopenia and obesity are also critically needed to determine and alter the trajectories of developing dementia in these groups.

## Data Availability

Publicly available datasets were analyzed in this study. This data can be found at: https://charls.charlsdata.com/.

## Footnotes

^1^ http://charls.pku.edu.cn/en
